# Supplementary material for: The feasibility analysis of integrating community-based health insurance schemes into the national health insurance scheme in Uganda
Source: PLoS One. 2023 Apr 14;18(4):e0284246. doi: 10.1371/journal.pone.0284246 (PMC10104299; doi:10.1371/journal.pone.0284246)
Supplement: S1 Table — (DOCX) [file pone.0284246.s001.docx]

Additional File 1: Study districts, CBHIS types and service providers

| **District** | **CBHI Type** | **Service Provider** |
| --- | --- | --- |
| Bushenyi | Provider Managed | Katungu Mission Hospital |
| Rukiga | Provider Managed | Kitanga HC III |
| Nakaseke | Community Managed | Kiwoko Hospital |
| Mubende | Community Managed | St. Gabriel Mirembe Maria HCIII |
| Rukungiri | Provider Managed | Kisiizi Hospital |
| Luwero | Third Party Managed | Bishop Ceasar Asili Hospital |
| Masaka | Third Party Managed | Kitovu Hospital |
