# Supplementary material for: The feasibility analysis of integrating community-based health insurance schemes into the national health insurance scheme in Uganda
Source: PLoS One. 2023 Apr 14;18(4):e0284246. doi: 10.1371/journal.pone.0284246 (PMC10104299; doi:10.1371/journal.pone.0284246)
Supplement: S2 Table — (DOCX) [file pone.0284246.s002.docx]

Additional file 2: List of Key Informants

| **Category** | **Number of respondents** |
| --- | --- |
| Scheme managers or technical staff of CHIs | 7 |
| Scheme chairpersons | 5 |
| Healthcare providers | 5 |
| UCBHFA Secretariat | 1 |
| CHI Promoters (SHU, medical bureaus, Health Partners Uganda) | 3 |
| Ministry of Health officials (CHS (PFP), NHIS secretariat) | 2 |
| Ministry for Finance | 2 |
| Development Partners (WHO, World Bank) | 2 |
| Ministry for Gender, Labour and Social Development | 1 |
| DHOs (including Head of DHOs Association) | 2 |
| District Community Development Officer | 1 |
| CSOs | 2 |
| **Total** | **33** |
