# Supplementary material for: The feasibility analysis of integrating community-based health insurance schemes into the national health insurance scheme in Uganda
Source: PLoS One. 2023 Apr 14;18(4):e0284246. doi: 10.1371/journal.pone.0284246 (PMC10104299; doi:10.1371/journal.pone.0284246)
Supplement: S3 Table — (DOCX) [file pone.0284246.s003.docx]

Additional file 3: Experiences about the Integration of CBHI into NHIS: Rwanda, United Republic of Tanzania, and Ghana

| 1. **RWANDA** | |
| --- | --- |
| **Overview** | Rwanda is one of the few African countries to successfully implement the CBHI program as an integral part of its national healthcare financing system with big coverage. |
| **The objective of the integration** | In Rwanda, the integration aimed to build a strong foundation for universal health coverage by consolidating the management of pension and insurance schemes, improving the efficiency and financial accountability of CBHIS, and reducing administrative costs. |
| **What influenced the integration?** | Integration in Rwanda was influenced by the emergence of universal access to health on the global agenda. Prior to 2015, CBHI schemes were managed independently and served specific health facility catchment area populations without an adequate option for healthcare referrals. Integration was needed to improve pooling as well as address structural inefficiencies and to cater for referrals. There was also a need for separation of responsibilities between institutional players where the Ministry of Health would be responsible for policy formulation and service provision and RSSB for policy implementation and purchasing. |
| **Current status**  **and practice** | **Pooling:** Pooling is at different levels: (i) CBHI branches (financing sources - members’ contributions, subsidies for the poor and other vulnerable people from Government and development partners); (ii) District (financing sources include national risk pooling, CBHI branches -45% of members' contributions, Government and development partners); (iii) National **(**financing sources include Government, Social health insurance (RAMA, MMI), private health insurance, development partners, CBHI district risk pooling - 10% of the 45% coming from CBHI branches). There are three levels of premiums. Category 1- (*people living in abject poverty*) member premiums are paid by the government and Category 2 (*very poor people*) and 3 (*Poor*) member premiums are paid directly by the members to their respective CBHI branches. Payment is due at the beginning of the Rwandan fiscal year (July-June) and covers membership during the entire year. There is flexibility for the collection of premiums whereby households can pay by instalments. If a person joins in the first month (July), there is no waiting period. However, a one-month waiting period is applied to those who join after that month. A flat co-payment fee (RWF 200 equivalent to USD 13) for each visit is applied at the health center level and 10 percent of the total hospital bill is collected from the patient as co-payment (CBHI Category I patients are exempted). Co-payments are collected at the health center level by CBHI branches and retained by the section to help cover CBHI administration costs. Hospital co-payments are collected and retained by the hospital.    **Purchasing**   - **Benefit package:** At the health center level: all medical services are specified in the minimum package of activities as defined by the MOH and ambulance bills. For CBHI members these services are paid by CBHI branches. At the district hospital, the CBHI at the district (district-level risk pooling) pays for the complementary package of activities and ambulance bills. At the referral hospital level, the services provided under the tertiary package of activities as well as ambulance bills are covered by the national pooling risk. - **Provider payment mechanisms:** Both the health centers and hospitals submit itemized, fee-for-service bills to the CBHI branches and district offices for reimbursement. At the hospital level, these bills can have as many as 20 items (consultation, hospitalization, tests, procedures, medicines, and medical supplies). Since many hospitals do not have sufficient financing, there may be some pressure to over-prescribe services or to over-bill. - **Provision:** Government remains the main provider of health services at health centers, district hospitals, and tertiary hospitals. |
| **Challenges** | Cross district reimbursement may still be an issue that needs to be addressed especially in the light of the new policy allowing patient roaming services. The financing shortfalls have been covered by contributions from the government and partners although it can take considerable time before that funding arrives at the CBHI. Overbilling has been reported in some districts. However, there are no identified benefits to hospital staff, and as such unlikely to be widespread. There are also often long delays in the reimbursement of CBHI bills which affects service delivery at health facilities. |
| 1. **THE REPUBLIC OF TANZANIA** | |
| **Overview** | Tanzania currently has multiple health insurance schemes; the proposed single national health insurance scheme was shelved |
| **The objective of the integration** | The integration into a single NHIS sought to end the fragmentation of health insurance coverage, increase resources for health, provide a minimum benefits package for all, and increase the efficiency of health spending. |
| **What influenced the integration?** | The idea of a single national health insurance scheme (SNHIS) was driven by a policy coalition of bureaucrats and transnational actors, inspired by shifts in international policy paradigms. In particular, this was influenced by the emergence of universal access to health on the global agenda. |
| **Current status**  **and practice** | Following the initial rejection of a single national health insurance scheme in December 2017, Tanzania currently has multiple health insurance schemes—including a social security fund, a private insurance option, a community-based insurance program, as well as insurance schemes for both the informal sector and formal public sector. Collectively, population coverage among all of these schemes is approximately 15 percent. According to the MOHSW, the fragmented insurance schemes have not reached a sufficient scale of proportional coverage among the population. |
| 1. **THE REPUBLIC OF GHANA** | |
| **Overview** | In Ghana, the integration has been successful: over 145 disjointed DMHHIS and the voluntary MHOs have consolidated into a single national health insurance system |
| **The objective of the integration** | The integration aimed to establish a national system to provide health coverage for the majority of the population |
| **What influenced the integration?** | Integration in Ghana was majorly influenced by politics. electoral campaigns of 1999 focused on promises to abolish “cash and carry” and the establishment of health insurance. Upon winning elections in December 2000, the opposition party (NPP) after winning the elections in December 2000, embarked on fulfilling its manifesto promise of improving health insurance coverage |
| **Current status**  **and practice** | - The individual MHOs or their network were forced to suspend their operations or merge with new district schemes. - Over 145 disjointed DMHIS and the voluntary MHOs were consolidated into a single unified payer NHIS. This implied that the scheme had one administrative system, one governance, and the power to accredit service providers (Implication, the integration of CHIs can be down gradually). - The DMHIS receives a subsidy from National Health Insurance Council (NHIC). - NHIA licenses and regulates the DMHIS, which each District Assembly is required to help establish by the NHI Act. (District assemblies are district-level government authorities, with locally-elected and presidentially-appointed representation). The governance of DMHIS was initially intended to be by locally-elected boards and managers (following the spirit of MHOs) it was slowly centralized and since 2008, local boards were disbanded. - Upon integration, the Ghana NHIA established a functioning administrative structure with a world-class head office complex. It now has 17 different. Also, it has 10 regional offices and over 130 district offices within the country. - Contrary to the MHO model, the NHIS is financed on a national basis from a single national risk pool in the National Health Insurance Fund (NHIF). The revenue sources for this fund include NHI levy (VAT), Deductions from payrolls from Social Security and National Insurance Trust (SSNIT), NHIF investment income, donor funds, and from premiums of the informal sector. |
| **Challenges** | - Costs and leakages to the scheme is the main challenge facing Ghana’s NHIS. The key drivers threatening the financial sustainability of the scheme include provider and insured moral hazard, adverse selection, and fraud. Others include increased enrolment which results into increase claims management, administrative costs and leakages, and huge premium exemptions. Also, lack of capacity development and managerial abilities – even with the constant trainings. |
