# Supplementary material for: The feasibility analysis of integrating community-based health insurance schemes into the national health insurance scheme in Uganda
Source: PLoS One. 2023 Apr 14;18(4):e0284246. doi: 10.1371/journal.pone.0284246 (PMC10104299; doi:10.1371/journal.pone.0284246)
Supplement: S4 Table — (DOCX) [file pone.0284246.s004.docx]

Additional file 4: Coverage and current enrolment for CBHIS in Uganda

| **Type of scheme** | **Scheme name** | **Current**  **enrolment** | **Geographical coverage by district** |
| --- | --- | --- | --- |
| **Third party managed** | 1. Save for Health -SHU-CHI Scheme | 522 | Luwero, Nakasongola and Masaka |
|  | 1. ICOCARE Community Health Plan for All | 579 | Sheema |
| **Community managed** | 1. Munno Mu Bulwadde Union of Schemes Organisations (MBUSO) | 26,087 | Luwero, Nakaseke and Nakasongola |
|  | 1. Western Ankore Tweragurize Scheme Organisation | 2,838 | Bushenyi, Sheema and Mitooma |
|  | 1. Munno Mu Bulwadde Cooperation Mechanism | 2,276 | Kassanda/Mubende |
|  | 1. Ishaka Health Plan | 2,500 | Bushenyi, Sheema, Mitooma, Buhweju and Rubirizi |
| **Provider managed** | 1. Kisiizi Hospital CBHI | 43,615 | Rukungiri, Ntungamo, Rukiiga, Kanungu and Kabale |
|  | 1. E- Quality | 28,032 | Kanungu |
|  | 1. Kuluva Hospital schem | 1,400 | Arua |
|  | 1. West Ankole Diocese Health Cooperative | 11,325 | Buhweju, Bushenyi, Sheema, Mitooma andRubirizi |
|  | 1. Uganda Health Cooperative | 2,517 | Bushenyi and Mitooma |
|  | 1. Ankole Diocese Health Cooperative | 1,754 | Mbarara, Isingiro,  Ntungamo, Kiruhura, Ibanda,Bushenyi, Sheema, and Kiruhura |
|  | 1. Arch Diocese of Mbarara | 2,986 | Mbarara, Isingiro, Ntungamo, Kiruhura, Ibanda, Bushenyi, Sheema and Kiruhura |
|  | 1. Kagando Hospital scheme | 6,757 | Kasese |
|  | 1. Buhweju People’s Health Cooperative | 2,628 | Buhweju and parts of Sheema |
|  | 1. Diocese of Kigezi Health Cooperative | 1,122 | Kabale, Rukiga and Rubanda, |
|  | 1. Kadoth reproductive and maternal health scheme | 1,250 | Rukungiri |
|  | 1. St. Luke Bujuni and St. Zoromina and Joseph Kibaale scheme | 1,211 | Kibaale |
|  | 1. Alleluyah joint maternity scheme | 494 | Alebtong, Abim |
|  | 1. Aero Medical Centre scheme | 64 | Oyam, Kole |
|  | 1. Bishop Ceaser Asilli hospital pre-health cooperative | 336 | Luwero |
|  | 1. Comboni CHI Scheme | 1,524 | Bushenyi |
|  | 1. Faith Mulira HCIII scheme | 250 | Wakiso |
|  | 1. Karin HCIII scheme | 183 | Gulu |
|  | 1. St. James Masiriba Community Health Insurance scheme | 381 | Kiboga |
|  | 1. Kayoro HCIII Scheme | 415 | Tororo |
|  | 1. Nyakatare HC III pre-health coop | 795 | Kanungu |
| **Provider & community managed** | 1. Kabale Diocese CHI Scheme | 11,216 | Kabale, Kisoro, Rubanda, Kanungu, Rukiga and Rukungiri |
| **Average enrolment** | | **5,538** |  |
| **Total enrolment** | | **155,057** |  |
