# Supplementary material for: The feasibility analysis of integrating community-based health insurance schemes into the national health insurance scheme in Uganda
Source: PLoS One. 2023 Apr 14;18(4):e0284246. doi: 10.1371/journal.pone.0284246 (PMC10104299; doi:10.1371/journal.pone.0284246)
Supplement: S5 Table — (DOCX) [file pone.0284246.s005.docx]

Additional file 5: Example of different packages and ceilings from a third-party managed scheme

|  | **What is comprised in the benefit package** | **Ceiling Premium**  **Amount per year**  **(UGX)** | | |
| --- | --- | --- | --- | --- |
| **1.** | a) Family premium, for only old families (re-contributing) regardless of the number of people in the family. Co-payment is UGX 5000 for IPC and OPC. This is for just 1 facility HC (III or IV). You can only go up to a maximum of 5 times a year, if you exceed you repay UGX 630,000 | 63,000 | 150,000 |  |
|  | b) For a new family covering 1-6 people; Any additional person pays UGX 10,500, Co-payment – UGX 5000 for IPC and OPC with the ceiling at UGX 150,000. This is for just 1 facility HC (III or IV). You can only go up to a maximum of 5 times a year, if you exceed you repay UGX 630,000 |  |  |  |
| **2** | a) For old family no limit on the number  Co-payment: UGX 30,000, 2 facilities (HC III – UGX 100,000, H/C IV –UGX 150,000, hospital –UGX 200,000). You get up UGX 400,000 if you are referred  b)1-6 people in a new family; UGX 12,000 additional  Co-payment –UGX 30,000, 2 facilities (HC III - UGX100,000; H/C IV –UGX 150,000; hospital - UGX200,00). You get up UGX 400,000 if you are referred | 72,000 | 400,000 |  |
| **3.** | UGX 120,000 old family members regardless of people  UGX 120,000 for 1-6membes in a new family, each additional member pays UGX 20,000  Co-payment UGX 5000, (2 HCs, 1 Hospital) any HC but you only go to a Hospital at referral. Up to a maximum of 5 times for the card to expire. | 120,000 | 350,0000 |  |
| **4.** | UGX 156,000 - old family with all members  UGX 156,000 for 1-6people in a new family, UGX26, 000 for any additional member  Co-payment UGX 20,000 (2 facilities of any level) | 156,000 | 400,000 |  |
| **5.** | UGX 210,000 old families with all members  UGX 210,000 – 1-6 people for a new family, UGX 35000 for any additional member  Co-payment – UGX 10,000 (2 facilities of your choices and 2 facilities on referral) | 210,000 | 400,000 |  |
