# Supplementary material for: The feasibility analysis of integrating community-based health insurance schemes into the national health insurance scheme in Uganda
Source: PLoS One. 2023 Apr 14;18(4):e0284246. doi: 10.1371/journal.pone.0284246 (PMC10104299; doi:10.1371/journal.pone.0284246)
Supplement: S6 Table — (DOCX) [file pone.0284246.s006.docx]

Additional file 6: Functionality and Financial Sustainability Aspects of CBHIS

| **DIMENSION** | **PROVIDER MANAGED CBHI (N=3)** | **COMMUNITY MANAGED CBHI (N=2)** | **THIRD-PARTY MANAGED CBHI (N=2)** |
| --- | --- | --- | --- |
| 1. **FUNCTIONALITY OF GOVERNANCE AND LEGAL STRUCTURE** | | | |
| **Legal Requirements and Status** | The schemes work as quasi-departments of the facility which are registered with the Uganda Medical and Dental Practitioners Council. They all had by-laws that were updated as needed. | One scheme was fully registered with the district as a CBO. While the second scheme had applied for legal status, but it was yet legally recognized in the new district. | The schemes had an expired registration**.** |
| **Organizational Structure** | Out of the three schemes, 2 had well-documented organizational structures while one lacked any documented organizational structure. | The schemes had organizational structures that were documented but needed to be updated. The structures were followed and were based on reasonably clear roles and responsibilities. | There was a documented organizational structure. However, it required some updating. It was not usually followed |
| **Board Composition and Responsibility:** | The external boards had members drawn from a broad spectrum. They were independent of management. The boards held regular and documented meetings at least thrice a year with adequate quorum. The boards had well-defined term limits. There were open and transparent procedures for electing or appointing and removing members and officers. | The boards were comprised of members drawn from a reasonably broad spectrum. They are independent of management. Regular and documented board meetings were organized. The boards have term limits. | The scheme external boards:   - Members are drawn from a narrow group - Limited independence from management - A written term of reference - The boards’ term limits were defined. |
| 1. **FINANCIAL MANAGEMENT, STRATEGIC PLANNING, AND SUSTAINABILITY** | | | |
| **Ability to budget and plan financial resources** | - Only one scheme had a clear standalone r budget; revenues and expenditures were monitored against the budget. - The second scheme had its budget integrated into one of the hospitals while the third scheme lacked realistic, clear, and well documented; revenues (premiums, donations etc) and expenditures are not monitored against budgets. | - The schemes had specific budgets - The scheme’s budgets were reasonably realistic, clear, and documented without significant external assistance - Revenues and expenditures were monitored against budgets monthly | - The schemes had a master budget, which is reasonably realistic, clear, and documented - Revenues and expenditures were being monitored against the budget. |
| **Availability of qualified finance team** | The schemes lacked registered accountants but rely on the hospital finance staff | No registered accountants but rely on the promoters’ accountants | The schemes had registered accountants. However, there were no clear job descriptions |
| **Financial Audit** | - The 2 schemes were audited as part of the hospitals - There were no regular financial audits for the past three years for the third scheme. | - Regular audit report for the past three years and management actions addressed - Audit report reflected in the work | - Regular financial audits were being conducted. |
| 1. **STRATEGIC PLANNING AND SUSTAINABILITY: RESERVES, GUARANTORS, AND RE-INSURANCE PLANS** | | | |
| **Sustainability mechanism of the scheme** | The schemes   - Are guaranteed by the hospital - No reserves - No re-insurance | The schemes had:   - Inadequate reserves and no re-insurance | The scheme had inadequate reserves; no guarantors and no re-insurance |
| 1. **STRATEGIC PURCHASING** | | | |
| **Strategic purchasing** | The schemes had:   - A defined benefit packages - No existence of gate-keeping - No agreed-on pricing of services | The schemes had:   - A defined benefit packages - A greed-on pricing of services - Existence of gate-keeping | The schemes had: A defined benefit packages and agreed-on pricing of services. |
