# Supplementary material for: The feasibility analysis of integrating community-based health insurance schemes into the national health insurance scheme in Uganda
Source: PLoS One. 2023 Apr 14;18(4):e0284246. doi: 10.1371/journal.pone.0284246 (PMC10104299; doi:10.1371/journal.pone.0284246)
Supplement: S7 Table — (DOCX) [file pone.0284246.s007.docx]

Additional file 7: Proposed stakeholders and roles

| **Stakeholder** | **Role** |
| --- | --- |
| Government (MOH) | - Regulation - Provision of health education and disease prevention |
| CSOs (They are community representatives) | - Advocating for their inclusion in the NHIS - Monitoring and asking for accountability from the fund manager - Can also get involved in enrolling members into NHIS - General mobilization of communities |
| Media | - Popularizing the scheme and integration - Educating the masses about the benefits of health insurance (especially on the integration of CBHIS) |
| Committee of Parliament on Health | Can ensure that MOH passes regulatory instruments on the integration of CBHI in NHIS |
| The parliamentary platform on social protection | They can help champion the integration of the flow of parliament |
| Ministry of Gender, Labour and Social Development | - Can do social advocacy for this integration - Support the MoH to categorize the indigents and other segments who cannot fully pay the actual premium |
| Special interest groups like women & elderly | They are likely to advocate for this because their constituencies are mainly in the informal sector and the most affected (they can start demanding and advocacy start from them). |
| Ministry of Finance, Planning, and Economic Development | - Can support in modelling what government can be able to contribute if there is any subsidy, studies on the benefits of this to the economy, how it will contribute to growth etc - Mobilize funds and allocate them especially for subsidizing the premiums. |
| Districts and other local governments) | The CBHIs are in their districts they can provide space (office), can do mobilization, support the CBHI structures and their members, promoting CBHIS. |
| Ministry of Gender, Labour and Social Development and Equal Opportunities Commission | - Key in supporting the policy of social protection – including CBHIs from the start. - They want people to access services, so they could advocate for it. |
