# Supplementary material for: The feasibility analysis of integrating community-based health insurance schemes into the national health insurance scheme in Uganda
Source: PLoS One. 2023 Apr 14;18(4):e0284246. doi: 10.1371/journal.pone.0284246 (PMC10104299; doi:10.1371/journal.pone.0284246)
Supplement: S8 Table — (DOCX) [file pone.0284246.s008.docx]

Additional file

**Capacity assessment tool of CBHI schemes in Uganda**

| **SCORING** | **INADEQUATE CAPACITY (1)** | **WEAK CAPACITY**  **(2)** | **ADEQUATE CAPACITY**  **(3)** | **STRONG CAPACITY**  **(4)** |
| --- | --- | --- | --- | --- |
| **1. Functionality of Governance and Legal Structure** | | | | |
| **1.1 Legal Requirements and Status** | | | | |
| **Subsection Objectives:** Determine the organization’s legal registration and compliance with national and local laws  **Documents:** Registration documents, constitution, articles of incorporation, and by-laws. Etc. | The scheme has   - Not legally registered or has an expired registration - No by-laws | The scheme has   - Applied for legal status, but is not yet legally recognized - By-laws, but they are incomplete or out of date. | The scheme has   - Legally registered and obtained all required permits and licenses - by-laws that are appropriate, but may need some updating | The scheme has   - Legally registered and is in fully compliance with all required permits and licenses - By-laws that are updated as needed |
| **1.2. Organizational Structure** | | | | |
| **Objectives:** Assess whether the organizational structure is appropriate **Subsection Resources:** Organization chart (organogram) or description of the staffing pattern; senior manager and staff questionnaires or interviews | The organizational structure is   - Informal or undocumented - Documented, but not or followed (not all positions are filled) - Based on inadequately defined departmental or functional responsibilities and lines of authority | The organizational structure is:   - Documented, but incomplete Not filled - Not usually followed - Based on partially clear roles and responsibilities of departments or functions and lines of authority | The organizational structure is   - Documented and adequate, but may require some updating - Usually followed - Based on reasonably clear roles and responsibilities of departments or functions and lines of authority | The organizational structure is   - Documented, good and updated as needed - Consistently followed - Based on well-defined roles and responsibilities of departments or functions and lines of authority |
| **1.3 Board Composition and Responsibility** | | | | |
| **Objectives:** Assess the board’s composition, terms of reference, procedures and oversight  **Subsection Resources:** Board membership list; description of board responsibilities, minutes of board meetings. | An external board has   - Not been established or is not functional - Little or no independence from management - No regular meetings - No term limits - No process for electing or appointing and removing members and officers - No written terms of reference or does not understand its functions   Only an advisory role | The external board has   - Members drawn from a narrow group - Limited independence from management - Regular meetings less than twice a year - Meetings that are not well attended - Ineffective or poorly documented meetings - A weak written term of reference or limited understanding of its functions - Term limits that are not defined or reasonable - No process for electing or appointing and removing members and officers | The external board has   - Members drawn from a reasonably broad spectrum - Moderate independence from management - Regular meetings at least twice a year - Meetings that consistently have a quorum - Reasonably effective and adequately documented meetings - An adequate written term of reference and understanding of its function - Reasonable, defined term limits - A process for electing or appointing and removing members and officers | The external board has   - Members drawn from a broad spectrum - Good independence from management - Regular meetings at least three times a year - Meetings attended by all or nearly all members - Effective and well documented meetings - A good written term of reference and understanding of its functions - Reasonable, defined term limits - Open and transparent procedures for electing or appointing and removing members and officers |
| 1. **Financial management, Strategic planning and Sustainability** | | | | |
| **2.1 Budgeting** |  |  |  |  |
| **Subsection Objectives:** Assess the ability to budget and plan financial resources  **Documents:** Annual and multi-year budgets, financial policies and procedures manuals, financial monitoring tools, revenue and expenditure reports, and financial staff questionnaires or interviews | - There is no master budget for the scheme - The scheme budgets are not realistic, clear, and well documented - Indirect costs are not calculated or are based on an inadequate methods or data - Revenues (premiums, donations etc) and expenditures are not monitored against budgets | - There is a master budget - The scheme budgets are only realistic, clear, and well documented with external assistance - Indirect costs are calculated with external assistance or are based on weak methods or data - Revenues and expenditures are monitored against budgets quarterly | - There is a master budget - The scheme budgets are reasonably realistic, clear, and documented without significant external assistance - Indirect costs are calculated without external assistance and based on adequate methods and data - Revenues and expenditures are monitored against budgets monthly | - There is a master budget - The scheme budgets are realistic, clear, and well documented without external assistance - Indirect costs are calculated without external assistance and based on good methods and data - Revenues and expenditures are monitored against budgets monthly |
| **2.2 Finance staff** | | | | |
| **Subsection Objectives:** Assess whether there is a qualified finance team **Documents:** Organisational chart, Job description & CVs | - No qualified accountants - No job descriptions | - There are accountants but not yet completed professional training - Job descriptions exist, not clear and not followed | - There are qualified accountants - Job descriptions exist, but not followed | - There are qualified accountants - Clear job descriptions exist and followed |
| **2.3 Financial Audit** |  |  |  |  |
| **Subsection Objectives:** Assess whether scheme funds are audited  **Resources:** Audit reports, Work plan, budget | - No audit report for the past three year - Audit report not reflected in the work plans | - No regular audit report for the past three year - Audit report not reflected in the work | - Regular audit report for the past three year and management actions not addressed - Audit report reflected in the work | - Regular audit report for the past three year and management actions addressed - Audit report reflected in the work |
| **2.4 Strategic planning and sustainability: reserves, guarantors and re-insurance plans** | | | | |
| **Subsection Objectives:** Assess whether the sustainability mechanism of the scheme **Documents:** Strategic plan, Letter from Guarantors, Contracts with re-insurers | The scheme has   - No reserves of the scheme - No guarantors - No re-insurance | The scheme has   - Reserves of the scheme but no guarantors and re-insurance | The scheme has   - Reserves of the scheme and guarantors and no re-insurance | The scheme has   - Reserves of the scheme - Guarantors - Re-insurance |
| 1. **STRATEGIC PURCHASING** | | | | |
| **Subsection Objectives:** Assess whether the scheme carries out strategic purchasing  **Documents:** Insurance policy | The scheme has   - No defined benefit package - No existence of gatekeeping - No agreed-on pricing of services or provider payment mechanism | The scheme has   - A defined benefit package but no agreed-on pricing of services - No existence of gatekeeping | The scheme has   - A defined benefit package - A greed-on pricing of services - No existence of gatekeeping | The scheme has   - A defined benefit package - A greed-on pricing of services - Existence of gatekeeping |
