# Supplementary material for: The feasibility analysis of integrating community-based health insurance schemes into the national health insurance scheme in Uganda
Source: PLoS One. 2023 Apr 14;18(4):e0284246. doi: 10.1371/journal.pone.0284246 (PMC10104299; doi:10.1371/journal.pone.0284246)
Supplement: Attachment — Submitted filename: Response to Reviewers.docx [file pone.0284246.s010.docx]

**Response to reviewers’ comments**

We thank the review team for the additional useful comments which have greatly improved the manuscript. We have responded point by point to every item raised and put replies in a table indicating where the text has been inserted. The manuscript has been revised using track changes and a clean version of the manuscript has been provided.

| **Comment** | **Response** | **Where inserted** |
| --- | --- | --- |
| **Academic Editor** |  |  |
| Please ensure that your manuscript meets PLOS ONE's style requirements, including those for file naming. The PLOS ONE style templates | This comment has been fully taken on board. |  |
| "PLOS requires an ORCID iD for the corresponding author in Editorial Manager on papers submitted after December 6th, 2016. Please ensure that you have an ORCID iD and that it is validated in Editorial Manager. To do this, go to ‘Update my Information’ | This comment has been fully taken on board. |  |
| We note that you have indicated that data from this study are available upon request. PLOS only allows data to be available upon request if there are legal or ethical restrictions on sharing data publicly. For more information on unacceptable data access restrictions, please see | The statement has been revised to “All relevant data are within the paper and its supporting information files”. |  |
| Thank you for providing information regarding the report on the Ugandan Ministry of Health website from which your study was drafted. Please include your statement regarding this matter from the cover letter in the Methods section of the manuscript | Text added as the last sentence under data management “This manuscript was drafted from a study that was commissioned by the Ugandan ministry of health whose report is available on its website”. |  |
| **Reviewer #1** |  |  |
| **ABSTRACT**  **Background** - I would suggest you change the statement " where the rich will subsidize the treatment of the poor and the healthy will subsidize the treatment of the sick, young and elderly" to "where the rich will subsidize the treatment of the poor, the healthy will subsidize the treatment of the sick, and young will subsidize the treatment of the elderly". | Statement revised as suggested | Abstract under background sub-section |
| **Method** - This is a cross-sectional study with qualitative data collection method and review of documents. The study design stated here is questionable? Some of the data collection methods mentioned such as questionnaires, observations and archive were not performed or reported in the study. | The data collection methods mentioned that were not performed such as questionnaires, observations, and archives or reported in the study have been deleted from the text to reflect what was actually carried out but not planned. | Study design |
| **Result** - Give the USD equivalent of the UGX 75,215 | (USD 20.3) has been inserted in the text after the UGX figure | Abstract under findings sub-section |
| **Conclusion**- How did the authors arrive at this conclusion? The result presented in the abstract did not show this. | The text has been revised to read “The results demonstrate the possibility and provide a pathway to integrating CBHIs into the proposed NHIS. We however recommend implementation in a phased manner including first providing technical assistance to the existing CBHIS at the district level to address the critical capacity gaps. This would be followed by integrating all three elements of CBHIS structures. The last phase would then involve establishing a single fund for both the formal and informal sectors managed at the national level”. | Abstract under results sub-section |
| **TEXT** **General comments** - The write-up seems too lengthy. There are minor grammatical errors that need to be corrected. Abbreviations should be used in full the first time, before their subsequent use. The United States Dollar equivalent of the money stated within the text should be included. | The authors have rigorously proofread the manuscript to correct all the grammatical and sentence construction errors. Attempts have been made to shorten the manuscript without grossly affecting the paper’s content, especially by removing redundant unnecessary text. The comments about Abbreviations and USD equivalent have been fully taken on board. | Mainly the results and discussion sections. |
| **Introduction** - The authors gave a good background on the topic, showed the gap in knowledge and stated the aim of the study. | We acknowledge this compliment. |  |
| **Methods** **Study settings-** How were the districts selected? What sampling method was used? Please give details of the sampling technique. | The text has been revised as “We purposively selected districts that were covered by at least one of the three CBHIS typologies”. | Sub-section on study settings |
| **Data collection methods-** 1. I would suggest the first statement in this subsection is moved to the study design 2. Who were the questionnaires administered to? How was the data from the questionnaire analyzed? 3. What was observed? where were the observations carried out? 4. What do you mean by archive as a method? 5. All I saw reading through the methods were Reviews of literature and other documents, Key Informant Interviews and Secondary data analysis | The sentence was moved, and it is now the last part of the study design paragraph.  The key informant guide was administered to policymakers and implementers both at the district and national levels, scheme leadership, and members of civil society  The data collection methods mentioned that were not performed such as questionnaires, observations, and archives or reported in the study have been erased from the text to reflect what was actually carried out but not planned. | Study design |
| **RESULT AND DISCUSSION** These were merged in the write up I would suggest the authors separate the results from the discussion | A new section on the discussion has been created | Discussion |
| Lessons learnt how did you come about some points such as "Another important lesson is that long distances and high costs of transport to healthcare providers undermine enrolment. Some people were not joining CBHIS whose service providers were perceived to be far especially if they had alternatives" and "Furthermore, we learned that some families fail to pay the premiums when they have conflicting interests, especially in school fees payments and festival seasons coinciding with premium payments. Whenever CBHI renewal periods overlap with school fee periods, it affects retention in CBHIs". The result section did not mention these points as findings of the study but were raised as part of lessons learns | This section was part of the information that was collected so it has been moved to the results section | Last paragraph of the result section |
| **CONCLUSION** Paragraph 6, line 4 - I would suggest you change the statement " where the rich will subsidize the treatment of the poor and the healthy will subsidize the treatment of the sick, young and elderly" to "where the rich will subsidize the treatment of the poor, the healthy will subsidize the treatment of the sick, and young will subsidize the treatment of the elderly".  Other comments are within the manuscript | This comment has been fully taken on board. | Conclusion |
| **Reviewer# 2** |  |  |
| The paper provides a comprehensive account of Uganda's existing community-based health insurance schemes (CBHIS). It also examines the feasibility of integrating the CBHIS into a national health insurance scheme. I enjoyed reading the paper; very insightful. I particularly like how the authors relate Uganda's case to other sub-Saharan African countries (Ghana, Rwanda & Tanzania). I have a few minor comments, notwithstanding. | We warmly welcome this statement! |  |
| The **first** relates to the use of abbreviations. Please check all for consistency (e.g., should readers assume CBHIS, CBHI, CBHIs, CHBIS & CHBIs are used interchangeably? Also see DMHIS & DMHIs). Ensure all abbreviations are defined upon first use (see CHI, SACCOs, CBO). | This comment has been fully taken on board. | Through the document |
| **Second**, some sentences need to be checked. e.g.,  i. "The effort towards the creation of a national scheme comes at a time when there already existing CBHIS." (are missing?) ii. "Most of the reasons pointed to the need to accelerate achieving universal health coverage since the majority of the population." (Is that an incomplete sentence?) | This comment has been fully taken on board and read thus:  i)“The effort towards the creation of a national scheme comes at a time when there are already existing CBHIS”.  ii) “Most of the reasons pointed to the need to accelerate achieving universal health coverage for the majority of the population”. This is further elaborated in the subsequent quotes from key informants. | Result section |
| Third, I found it hard understanding the use of e.g. "1000/=". Please feel free to ignore this comment if it is a standard approach which I simply do not know. | Has been edited as Uganda shillings 1,000= | Results section |
