# Supplementary material for: The feasibility analysis of integrating community-based health insurance schemes into the national health insurance scheme in Uganda
Source: PLoS One. 2023 Apr 14;18(4):e0284246. doi: 10.1371/journal.pone.0284246 (PMC10104299; doi:10.1371/journal.pone.0284246)
Supplement: Attachment — Submitted filename: Response to Reviewers.docx [file pone.0284246.s011.docx]

**Response to reviewers’ comments**

We thank the review team for the second round of additional useful comments which have greatly improved the manuscript. We have responded point by point to every item raised and put replies in a table indicating where the text has been inserted. The manuscript has been revised for the second time using track changes and a clean version provided.

| **Comment** | **Response** | **Where inserted** |
| --- | --- | --- |
| **Journal Requirements** |  |  |
| Please review your reference list to ensure that it is complete and correct. If you have cited papers that have been retracted, please include the rationale for doing so in the manuscript text, or remove these references and replace them with relevant current references. Any changes to the reference list should be mentioned in the rebuttal letter that accompanies your revised manuscript. If you need to cite a retracted article, indicate the article’s retracted status in the References list and also include a citation and full reference for the retraction notice. | We have checked this through the document and replaced links that were not accessible. |  |
| Please upload your figure files to the Preflight Analysis and Conversion Engine (PACE) | This manuscript had no figure files. |  |
| **Academic Editor** |  |  |
| Questionnaire is an instrument of collecting data but not a method of data collection. Replace questionnaire with the appropriate method of data collection in the abstract | A survey as the data collection method has been inserted. The survey was conducted using a capacity assessment tool which has been provided as a supplement file. | abstract and methods section |
| All abbreviations used in the abstract should be written in full or defined within the abstract (eg: CHI and CBHI) | This comment has been fully taken on board and amendments made in the abbreviations section of the paper. |  |
| Please ensure that the manuscript is thoroughly proofread before resubmission | This comment has been fully taken on board and additional proofreading was done. |  |
| **Reviewer# 2** |  |  |
| Many thanks for addressing my concerns. It appears the paper still has "CHBIS". Please define upon first use or correct if you mean CBHIS. (see "Integration of CHBIS...") | This comment has been fully taken on board. |  |
